# Arkansas State Medical Society

**Published:** 1882-10-20

**Authors:** 


### *STATE MEDICAL SOCIETY OF ARKANSAS.*

Transactions of State Medical Society of Arkansas, held in Little Rock, May 31st, 1882, Seventh Annual Session:

A neat book of 163 octavo pages. It contains a list of the entire membership, and a list of all the officers during the several years of its existence, from the organization of the Society in 1875.

We note a number of interesting papers, to-wit:

The President's address, by R. G. Jennings, M. D., on State Legislation, State Board of Health, etc.

Address on Medical Education, by W. H. Hawkins, M. D.

Report of Committee on Medical Legislation, by Jas. H. Southall, M. D., Chairman.

A study of Red Blood Corpuscles, by T. E. Murrell, M.D.

Bilious Fever of Arkansas, by M. D. Cantrell, M.D.

Insanity and Insane Asylums, by P. O. Hooper, M.D.

Clinical Notes and observations on Prevailing Fevers, by J. A. Dibrell, Jr., M.D.

Plea for some neglected Branches in Medicine, by Geo. C. Hartt, M.D.

Malarial Poison, by Z. Orto, M.D.

Case of Obstruction of the Œsophagus from Scirrhus, by R. G. Jennings, M.D.

Nymphomania Cured by Operation for Hemorrhoids, by Wm. H. Hardison, M.D.

The Removal of an Impacted Calculus from the Urethra of a Child, by G. W. Hudson, M.D.

Treatment of Croup, with Hydrargeri Sulphas Flava; A Case of Ununited Fracture of Tibia and Fibula; by J. Bennett, M.D.

OFFICERS ELECT.

*President.*—J. H. Southall, M.D., Little Rock, Pulaski county.

*Vice Presidents.*—J. A. Dibrell, Sr., M.D., Van Buren, Crawford county; H. H. Turner, M.D., Ozark, Franklin county; Z. Orto, M.D., Walnut Ridge, Lawrence county; D. J. Prather, M.D., Prescott, Nevada county.

*Secretary.*—L. P. Gibson, M.D., Little Rock, Pulaski county.

*Assistant Secretary.*—Edward Meek, M.D., Argenta, Pulaski county.

*Treasurer.*— A. L. Breysacher, M.D., Little Rock, Pulaski county.

*Librarian.*—John Waters, M.D., Little Rock, Pulaski county.

The next meeting will be held at Little Rock, May 30th, 1883.

